# Adenosine A_2A_ Receptor Modulates the Activity of Globus Pallidus Neurons in Rats

**DOI:** 10.3389/fphys.2017.00897

**Published:** 2017-11-07

**Authors:** Hui-Ling Diao, Yan Xue, Xiao-Hua Han, Shuang-Yan Wang, Cui Liu, Wen-Fang Chen, Lei Chen

**Affiliations:** ^1^Department of Physiology, Qingdao University, Qingdao, China; ^2^Department of Physiology, Binzhou Medical University, Yantai, China; ^3^Department of Anatomy, Qingdao University, Qingdao, China

**Keywords:** globus pallidus, adenosine A_2A_ receptors, Parkinson's disease, extracellular single unit recording, elevated body swing test

## Abstract

The globus pallidus is a central nucleus in the basal ganglia motor control circuit. Morphological studies have revealed the expression of adenosine A_2A_ receptors in the globus pallidus. To determine the modulation of adenosine A_2A_ receptors on the activity of pallidal neurons in both normal and parkinsonian rats, *in vivo* electrophysiological and behavioral tests were performed in the present study. The extracellular single unit recordings showed that micro-pressure administration of adenosine A_2A_ receptor agonist, CGS21680, regulated the pallidal firing activity. GABAergic neurotransmission was involved in CGS21680-induced modulation of pallidal neurons via a PKA pathway. Furthermore, application of two adenosine A_2A_ receptor antagonists, KW6002 or SCH442416, mainly increased the spontaneous firing of pallidal neurons, suggesting that endogenous adenosine system modulates the activity of pallidal neurons through adenosine A_2A_ receptors. Finally, elevated body swing test (EBST) showed that intrapallidal microinjection of adenosine A_2A_ receptor agonist/antagonist induced ipsilateral/contralateral-biased swing, respectively. In addition, the electrophysiological and behavioral findings also revealed that activation of dopamine D_2_ receptors by quinpirole strengthened KW6002/SCH442416-induced excitation of pallidal activity. Co-application of quinpirole with KW6002 or SCH442416 alleviated biased swing in hemi-parkinsonian rats. Based on the present findings, we concluded that pallidal adenosine A_2A_ receptors may be potentially useful in the treatment of Parkinson's disease.

## Introduction

The rodent globus pallidus (homolog of the external segment of the primate globus pallidus) is a central nucleus in the indirect pathway of the basal ganglia circuit (Jellinger, 1991), which plays important roles in movement regulation under healthy and pathological states (Raz et al., 2000; Kita and Kita, 2001; Dodson et al., 2015; Hegeman et al., 2016). Based on the *in vivo* electrophysiological characteristics, the globus pallidus neurons are classified into three firing patterns including high-frequency without pauses, high-frequency with pauses and low-frequency with bursts (Benhamou et al., 2012). The spontaneous firing activities of pallidal neurons are closely associated with movement amplitude, velocity and direction (Gage et al., 2010; Hegeman et al., 2016). Previous studies have revealed that aberrant pallidal neuron activities appear at the onset and maintenance of motor dysfunction in Parkinson's diseases (Kita, 2007; Obeso et al., 2008). Furthermore, the decreased firing rate and synchronous bursting of pallidal neurons are strongly related to the motor symptoms of Parkinson's diseases (Raz et al., 2000; Sani et al., 2009; Chan et al., 2011).

Adenosine is an endogenous purine nucleoside which plays a wide variety of roles in central nervous systems, including development, sleep, synaptic transmission, pain, neuroinflammation, anxiety and depression. Adenosine receptors (A_1_, A_2A_, A_2B_, and A_3_) belong to G-protein-coupled receptors, with A_1_ and A_3_ receptors couple to Gi and Go while A_2A_ and A_2B_ receptors couple to Gs proteins (Fredholm et al., 2001, 2011; Ferre et al., 2008). It is known that adenosine A_2A_ receptors are highly expressed in the striatum, globus pallidus, nucleus accumbens, and olfactory tubercles of rat and human brain, as compared to other adenosine receptor subtypes with widespread brain distribution (Jarvis and Williams, 1989; Martinez-Mir et al., 1991; Rosin et al., 2003). The selective and specific location of adenosine A_2A_ receptors suggests that it may become a potential therapeutic target for basal ganglia diseases, particularly Parkinson's disease (Preti et al., 2015; Cunha, 2016). Considerable preclinical studies have shown that blockade of adenosine A_2A_ receptors could symptomatically relieve from parkinsonian motor deficits without L-DOPA-related motor side effects (Bibbiani et al., 2003; Jenner, 2014; Preti et al., 2015; Cunha, 2016).

Anatomical and morphological studies suggest that the adenosine A_2A_ receptors are principally expressed on striatopallidal terminals in the globus pallidus (Rosin et al., 2003; Shindou et al., 2003). Previous *in vitro* electrophysiological studies show that stimulation of adenosine A_2A_ receptors enhances GABA release and therefore augments the inhibitory postsynaptic currents (IPSCs) in the globus pallidus (Mori and Shindou, 2003; Floran et al., 2005). Other studies reveal that adenosine A_2A_ receptor activation exerts a dual effect on the release of GABA (Mayfield et al., 1993; Dayne Mayfield et al., 1996; Morales-Figueroa et al., 2014). However, Querejeta et al. (2010) demonstrate that intrapallidal infusion of adenosine A_2A_ receptor agonist and antagonist have no effects on the spontaneous firing rate of the globus pallidus neurons in both sham and ipsilaterally dopamine-denervated rats. As adenosine A_2A_ receptor antagonist is a potential therapeutic target for parkinsonian motor deficits (Bibbiani et al., 2003; Jenner, 2014; Preti et al., 2015; Cunha, 2016), the study of pallidal adenosine A_2A_ receptors will provide an insight into the movement regulation in both normal and parkinsonian states. Up to present, little is known about the *in vivo* electrophysiological and behavioral effects and the possible mechanisms of adenosine A_2A_ receptors in the globus pallidus under both normal and abnormal states. By using multibarrel microelectrode extracellular recordings and elevated body swing test (EBST), we therefore investigated the effects of adenosine A_2A_ receptors in the globus pallidus of both intact and 6-hydroxydopamine (6-OHDA) parkinsonian rats.

## Materials and methods

### Animals

Adult male Wistar rats (Qingdao, China), 8–10 weeks of age and weighing 220–290 g, were used in this experiment. Rats were housed in an environmentally controlled room at 22 ± 1°C with a 12 h light/dark cycle. The study was performed strictly in accordance to the University ethics guidelines. All operations were required to lower rats' suffering and pain. A total of 115 rats were used for electrophysiological experiments, with 81 normal rats and 34 successful parkinsonian rats. Three of the 37 parkinsonian rats did not show any stable recordings and were excluded from the study. In addition, 78 rats were used for behavioral study and 8 rats were used for immunoflurescence staining.

### Establishment of 6-hydroxydopamine (6-OHDA) hemi-parkinsonian rat model

Rats were injected with chloral hydrate (400 mg/kg, i.p.) and fixed in the stereotaxic instrument (NarishigeSN-3, Tokyo, Japan). 6-hydroxydopamine (6-OHDA, 4 μg/μl in saline with 0.01% ascorbic acid, 4 μl) was injected into the left medial forebrain bundle (AP −4.3, ML +1.7, DV −8.4 mm from Bregma) using microsyringe at a rate of 1.0 μl/min. Animals that exhibited at least 210 net contralateral rotations in 30 min after delivered apomorphine (0.2 mg/kg, s.c.) were considered as successful hemi-parkinsonism rats.

### Electrophysiological recordings *in Vivo*

One of the advantages of *in vivo* extracellular recording used in this study is to investigate the direct effects of drugs on the single pallidal neurons in both physiological and pathological conditions. The other advantage of the present multibarrel microelectrodes is to apply drugs directly to the neurons recorded. According to our previous studies (Xue et al., 2010; Chen et al., 2015), extracellular recordings were performed in the globus pallidus of normal urethane (1 g/kg, i.p.) anesthetized rats. Anaesthesia levels were monitored constantly by testing reflexes to a cutaneous pinch and maintained by giving supplement dose of chloral hydrate (0.1 g/kg, i.p.) when necessary. Rats were fixed in the stereotaxic instrument. Two holes were drilled bilaterally over the globus pallidus according to the stereotaxic atlas (0.8–1.2 mm posterior to bregma and 2.5–3.5 mm lateral to the midline, Paxinos and Watson, 1986). All exposed cortex were covered with a thin layer of saline to prevent drying. Recordings of neuronal spike and micro-pressure ejection of drugs were used with fined 3-barrelled micropipettes (tip diameter 3–10 μm, resistance10–20 MΩ). The recording microelectrode contained 2% pontamine sky blue dissolved in 0.5 M sodium acetate. The other two microelectrodes were filled with vehicle (saline) and various drugs, including CGS21680, KW6002, SCH442416, quinpirole, gabazine, nipecotic acid, or H-89. Drugs were delivered using 4-channel pressure injector (PM2000B, Micro Data Instrument, Inc., USA) with short-pulse gas pressure (1,500 ms, 5.0–15.0 psi). The recording electrical signal was amplified by a micro-electrode amplifier (MEZ-8201, Nihon Kohden, Tokyo, Japan), low- and high-pass filtered at 0.3 and 3 kHz, monitored with a memory oscilloscope (VC-11, Nihon Kohden, Tokyo Japan) and an audio monitor. The electrical signal was transferred into bio-electricity signal analyzer and computer. The pallidal spiking data capture and analysis were available via spike 2 software (Cambridge Electronic Design, UK).

Drug application was initiated after basal spontaneous firing of pallidal neurons remained stable for at least 10 min. The 120 s average firing before drug application was regarded as the basal firing, and the 50 s maximally changing discharge after drug administration was considered as the effect. The increase or decrease in firing frequency was considered statistically significant when the firing rate was higher or lower than the mean ±2 SD of the baseline (SD = standard deviation). Firing pattern was assessed via the coefficient of variation (CV) which referred to the standard deviation of the interspike intervals (ISI) divided by mean.

### Elevated body swing test (EBST)

EBST was performed in awake and freely-moving rats according to previously described methods (Borlongan et al., 1995; Baluchnejadmojarad and Roghani, 2004). The rat was placed in a transparent plastic cage (40 × 40 × 35 cm). After a 10 min-habituation, the rat was elevated to ~2 cm above the bottom of the cage by holding 2 cm above the bottom of tail. The body swing referred to the rat head with deviation of vertical axis more than 10°. Before every swing was recorded, the rat head must be suspended at a vertical axis. If the rat did not bend its head after being elevated over 5 s, the tail was gently pinched. Each test rat was recorded for 60 s. Initially, only rats showing unbiased behavior were chosen for the study.

All experimental rats were anesthetized with chloral hydrate (400 mg/kg, i.p.), and implanted with the stainless steel guide cannula in the globus pallidus on either side (o.d., 0.4 mm; i.d., 0.3 mm). The cannula was secured to the skull with screws and dental acrylic. The vehicle (saline) or drugs (CGS21680, KW6002, and SCH442416) was unilaterally microinjected into the globus pallidus with 1 μl microsyringe. The injection rate was maintained at 0.2 μl/min with a total volume of 0.5 μl and the microsyringe was kept in position for an additional 3 min before retraction.

The number of biased body swing was counted per minute. The percentage score was calculated for each rat, i.e., the number of biased swings was divided by the total number of swings and multiplied by 100%. Over 70% of biased swings were defined as the criterion for biased swing behavior.

### Double immunofluorescence staining

The double immunofluorescence technique was used to observe the expression of adenosine A_2A_ receptors and parvalbumin in rat globus pallidus. Brain tissue was obtained from normal and parkinsonian rats, and then was fixed in 4% paraformaldehyde overnight. Tissues containing the globus pallidus were sectioned at 40 μm after dehydration in 20 and 30% sucrose. Coronal globus pallidus sections were blocked with 5% donkey serum (Solaribo) and 0.3%Triton X-100 in PBS for 60 min at 4°C. And the sections were subsequently incubated with a rabbit polyclonal anti-adenosine A_2A_ receptor antibody (sc-13937, 1:100; Santa Cruz) and a goat polyclonal anti-parvalbumin antibody (ab3289, 1:500; Abcam) for 48 h at 4°C. After washing three times with PBS (5 min), the sections were incubated with the following secondary antibodies, Alexa 488-conjugated donkey anti-rabbit (1:1,000; Abcam) and Alexa 647-conjugated donkey anti-goat (1:1,000; Abcam), for 2 h at room temperature. Lastly, the sections were washed, mounted, coverslipped and examined under fluorescent microscope or laser scanning confocal microscopy (Leica, Wetzlar, Germany).

The results were analyzed by counting the number of adenosine A_2A_ receptor-positive cells in the globus pallidus. The adenosine A_2A_ receptor-positive cells were examined and quantified using Image J software (NIH, Bethesda, MD). We selected six sections (at the levels of 0.8–1.2 mm posterior to bregma) from the globus pallidus from each rat for cell counting. Results were expressed as the average number of positive cells obtained from the six sections (cells visualized at 400 × magnifications).

### Histological controls

**Figure 1:**
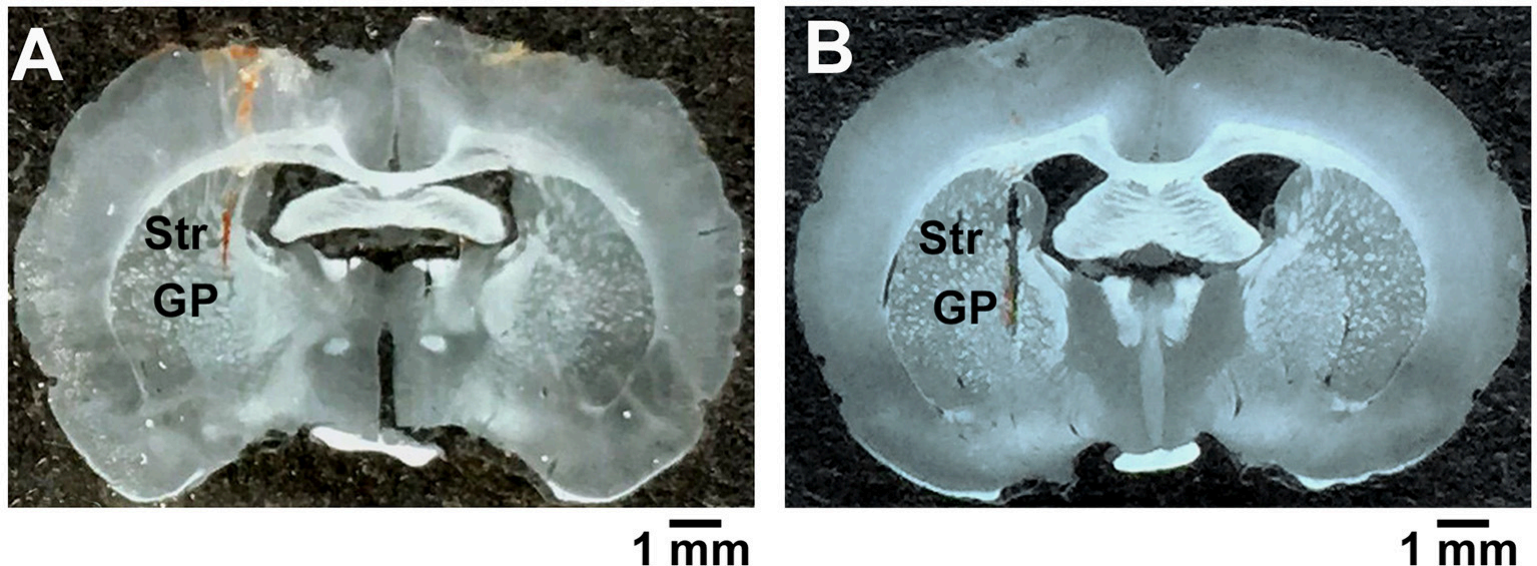
Confirmation of the recording and microinjection location within the globus pallidus. **(A)** A coronal brain section illustrating the trace of microelectrodes in the globus pallidus. **(B)** A coronal brain section showing the injector tip for microinjection. GP, globus pallidus; Str, striatum; scale bars = 1 mm.

After electrophysiological and behavioral experiments, we needed to verify the position of electrophysiological recording sites and cannula placements. Rats were deeply anesthetized and perfused transcardially with a saline solution containing 4% paraformaldehyde. Recording/microinjection sites were confirmed using camera (Figure 1). To further confirm the 6-OHDA parkinsonian rat model, coronal substantia nigra sections were incubated with a rabbit anti-tyrosine hydroxylase antibody (T8700, 1:1000; sigma) following secondary antibodies (1:1,000; Abcam) of Alexa 488-conjugated donkey anti-rabbit. The number of tyrosine hydroxylase-positive neurons on the lesioned side of the substantia nigra pars compacta decreased to 17.08 ± 1.84%, which was significantly lower than that of normal rats (**Figure 5A**).

### Drugs and statistical analysis

CGS21680, KW6002, SCH44216 and quinpirole were purchased from Tocris (Bristol, UK). DMSO, 6-OHDA hydrochloride, apomorphine hydrochloride, gabazine, and H-89 were obtained from Sigma (St Louis, Mo, USA). Nipecotic acid was purchased from Abcam (Cambridge, MA, USA).

All data were presented as mean ± S.E.M. Paired-samples *t*-test was used for comparing the difference of spontaneous firing before and after drug application. Statistical comparisons between or among groups were determined with independent-samples *t*-test and one-way ANOVA. Chi-square test was applied to compare the fractions of CGS21680 responsive neurons and different concentrations of drugs. Before *t*-tests, Levene's test F-test was performed to determine if the data is normally distributed. Wilcoxon–Mann–Whitney test was used for data that are not normally distributed. *P* < 0.05 was considered as the level of significance. Data analysis was performed using IBM SPSS 22.0 software.

## Results

### Effects of adenosine A_2A_ receptor activation on the spontaneous firing of globus pallidus neurons in normal rats

**Figure 2:**
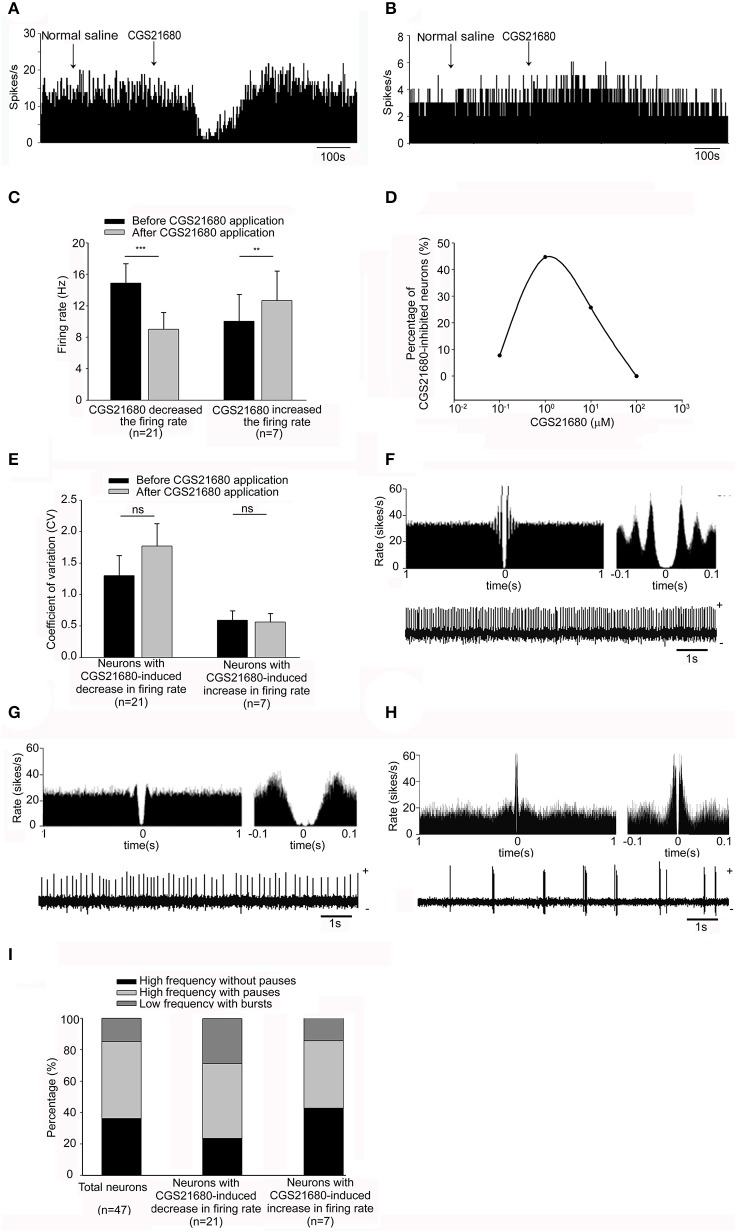
Effects of micro-pressure ejection of adenosine A_2A_ receptor selective agonist, CGS21680, on the spontaneous firing of pallidal neurons in normal rats. **(A)** Typical frequency histograms showing that 1 μM CGS21680 decreased the firing rate of a pallidal neuron. **(B)** In this neuron, 1 μM CGS21680 increased the firing rate slightly. **(C)** Pooled data summarizing the effects of 1 μM CGS21680 on the firing rate of pallidal neurons in normal rats. The black bars represent the basal firing rate before CGS21680 application, while the gray bars represent the firing rate after CGS21680 application. ^**^P < 0.01, ^***^P < 0.001, paired-samples *t*-test. **(D)** The graph summarizing the percentages of pallidal neurons with different concentrations (0.1, 1, 10, and 100 μM) of CGS21680-induced decrease in firing rate. **(E)** Pooled data summarizing the effects of CGS21680 on the CV of pallidal neurons. **(F–H)** Typical traces showing three firing patterns of globus pallidus neurons, high frequency without pauses **(F)**, high frequency with pauses **(G)**, and low frequency with bursts **(H)**. **(I)** Pooled data showing the proportion of the three firing pattern types in total neurons and CGS21680-induced inhibitory or excitatory neurons.

We first observed the effects of adenosine A_2A_ receptor selective agonist CGS21680 on the spontaneous firing rate of 47 pallidal neurons tested. The average neuronal firing rate was 14.02 ± 1.44 Hz. Micro-pressure administration of 1 μM CGS21680 significantly decreased the frequency of spontaneous firing from 14.89 ± 2.48 Hz to 9.03 ± 2.14 Hz in 21 out of the 47 pallidal neurons (*n* = 21, *P* < 0.001, *t* = 5.78, *df* = 20, paired-samples *t*-test, Figures 2A,C). The average decrease was 46.08 ± 5.71%, which was significantly different (*Z* = 4.72, *P* < 0.001, Mann-Whitney test) compared to that of vehicle administration (basal: 15.31 ± 3.24 Hz; vehicle: 15.58 ± 3.21 Hz; *n* = 12, *t* = 1.61, *df* = 11, *P* > 0.05, paired-samples *t*-test). In only 7 out of the 47 pallidal neurons, 1 μM CGS21680 increased the firing frequency from 10.05 ± 3.40 to 12.66 ± 3.77 Hz (*n* = 7, *t* = 4.34, *df* = 6, *P* < 0.01, paired-samples *t*-test, Figures 2B,C), with the average increase of 50.80 ± 20.68% (*Z* = 3.47, *P* < 0.01 compared to that of vehicle administration, Mann-Whitney test). In the remaining 19 pallidal neurons, 1 μM CGS21680 did not alter the firing rate significantly (*t* = 1.27, *df* = 18, *P* > 0.05, paired-samples *t*-test). Similar to that of 1 μM CGS21680, the higher concentration of CGS21680 (10 μM) also modulated the spontaneous firing of globus pallidus neurons. The average spontaneous firing rate of all the 35 pallidal neurons tested was 17.12 ± 2.24 Hz. Application of 10 μM CGS21680 decreased the frequency of spontaneous firing from 23.35 ± 5.90 to 13.93 ± 4.97 Hz in 9 out of the 35 pallidal neurons (*n* = 9, *t* = 6.94, *df* = 8, *P* < 0.001, paired-samples *t*-test). The average decrease was 47.70 ± 5.97%, which was significantly different (*t* = 8.73, *df* = 18, *P* < 0.001, independent-samples *t*-test) compared to that of vehicle administration (basal: 16.03 ± 2.83 Hz; vehicle: 16.42 ± 2.90 Hz; *n* = 11, *t* = 1.88, *df* = 10, *P* > 0.05, paired-samples *t*-test). CGS21680 at 10 μM increased the firing rate from 6.78 ± 2.82 to 9.91 ± 3.83 Hz in other 6 out of the 35 pallidal neurons (*n* = 6, *t* = 2.63, *df* = 5, *P* < 0.05, paired-samples *t*-test), with the average increase of 33.04 ± 1.60% (*t* = 10.99, *df* = 15, *P* < 0.001 compared to that of vehicle administration, independent-samples *t*-test). In the remaining 20 pallidal neurons, 10 μM CGS21680 did not alter the firing rate significantly. The highest concentration of CGS21680 (100 μM) only increased the pallidal firing rate in 3 out of the 19 neurons, while the lowest concentration of the drug (0.1 μM) only had decreasing effects in 1 out of the 13 neurons. Further analysis revealed that the percentages of pallidal neurons which were inhibited by different concentrations of CGS21680 (0.1, 1, 10, and 100 μM) were significantly different based on Chi-square test (*x*^*2*^ = 28.26, *df* = 6, *P* < 0.001). The percentage of reactive neurons (44.68%) with 1 μM CGS21680-induced inhibition of firing rate was strongest compared to that of other drug concentrations (Figure 2D), while there was no significant change in the percentage of reactive neurons with CGS21680-induced excitatory effects of all drug concentrations. Therefore, we chose the concentration of 1 μM to further explore the effects of adenosine A_2A_ receptors on pallidal neurons in the subsequent experiments.

Next, we analyzed the possible changes of firing pattern by calculating the CV before and after CGS21680 application. Micro-pressure ejection of 1 μM CGS21680 didn't change the CV significantly (basal: 1.30 ± 0.32; CGS21680: 1.77 ± 0.39; *n* = 21, *t* = 1.90, *df* = 20, *P* > 0.05, paired-samples *t*-test, Figure 2E) in the 21 neurons with CGS21680-induced decrease in firing rate. There was also no change of CV in the neurons with 1 μM CGS21680-induced increase in firing rate (basal: 0.59 ± 0.15; CGS21680: 0.56 ± 0.14; *n* = 7, *t* = 0.71, *df* = 6, *P* > 0.05, paired-samples *t*-test, Figure 2E). Similar to that of 1 μM CGS21680, there was no significant change in CV following micro-pressure ejection of CGS21680 at the concentrations of 0.1, 10, and 100 μM (data not shown).

According to our previous study (Chen et al., 2015), we classified pallidal neurons into three types (i.e., higher frequency without pauses, higher frequency with pauses and low frequency with bursts, Figures 2F–H). Based on this classification, the proportion of 36.17% high frequency without pauses, 48.94% high frequency with pauses and 14.89% low frequency with bursts were observed within the present 47 pallidal neurons. Furthermore, within 21 out of the 47 pallidal neurons with CGS21680-induced decrease in firing rate, these firing pattern types represented a proportion of 28.57, 47.62, and 23.81%, respectively. In another 7 CGS21680-induced excitatory neurons, 42.86, 42.86, and 14.28% exhibited above three firing patterns, respectively. There was no significant difference among the proportions of firing pattern types of the neurons in CGS21680-induced inhibitory or excitatory neurons and the total 47 neurons (*x*^2^ = 1.15*, df* = 4, *P* > 0.05, Chi-square test, Figure 2I).

### Endogenous adenosine modulated the spontaneous firing of globus pallidus neurons through adenosine A_2A_ receptors in normal rats

**Figure 3:**
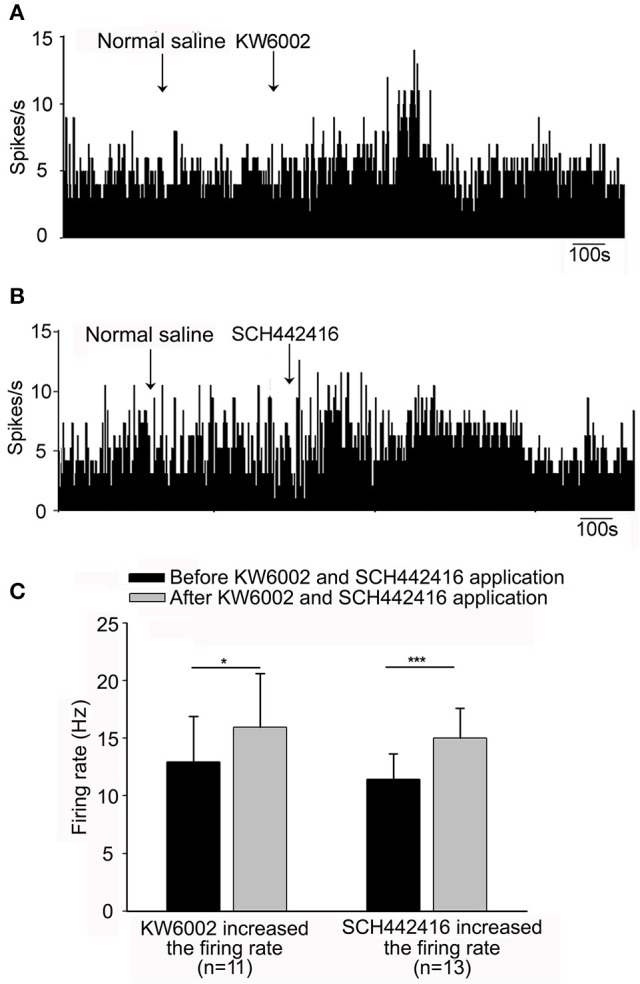
Effects of adenosine A_2A_ receptor antagonists, KW6002 and SCH442416, on the spontaneous firing of pallidal neurons in normal rats. **(A)** Typical frequency histograms showing that 1 μM KW6002 increased the firing rate of a pallidal neuron. **(B)** In this neuron, 1 μM SCH442416 increased the firing rate. **(C)** Pooled data summarizing the excitatory effects of both KW6002 and SCH442416 on the firing rate of pallidal neurons. The black bars represent the basal firing rate before drugs application, while the gray bars represent the firing rate after drugs application. ^*^P < 0.05, ^***^P < 0.001, paired-samples *t*-test.

To elucidate the possible modulation of spontaneous pallidal neuronal activity by endogenous adenosine through adenosine A_2A_ receptors, we explored the effects of selective adenosine A_2A_ receptor antagonists on the spontaneous firing activity of pallidal neurons. Micro-pressure administration of selective adenosine A_2A_ receptor antagonist, KW6002 (1 μM), significantly increased the spontaneous firing rate from 12.92 ± 3.98 to 15.95 ± 4.70 Hz in 11 out of the 27 pallidal neurons (*n* = 11, *t* = 3.05, *df* = 10, *P* < 0.05, paired-samples *t*-test, Figures 3A,C), with the average increase of 29.48 ± 10.76% (*Z* = 2.97, *P* < 0.05 compared to that of vehicle administration, Mann-Whitney test). In 4 neurons, 1 μM KW6002 decreased the firing rate from 9.81 ± 4.50 to 6.82 ± 3.67 Hz. In the remaining 12 neurons the firing rate was not affected (data not shown).

Based upon above results, the present study first demonstrated that endogenous adenosine modulated the spontaneous firing of pallidal neurons *in vivo*. To further provide more evidence, we continued to observe the effects of another selective antagonist SCH442416 on the pallidal firing. Similar to that of KW6002, the predominant effect of SCH442416 (1 μM) was an increase in the firing rate of globus pallidus neurons. Micro-pressure administration of 1 μM SCH442416 significantly increased the spontaneous firing rate from 11.46 ± 2.19 Hz to 15.03 ± 2.58 Hz in 13 out of the 22 pallidal neurons (*n* = 13, *t* = 6.03, *df* = 12, *P* < 0.001, paired-samples *t*-test, Figures 3B,C). The average increase was 38.78 ± 8.56%, which was significantly different (*Z* = 3.91, *P* < 0.001, Mann-Whitney test) from that of vehicle ejection. Moreover, we compared the increasing effects of two antagonists, SCH442416 and KW6002, on the spontaneous firing rate of pallidal neurons. The excitatory action of 1 μM SCH442416 on firing rate was slightly stronger than that of 1 μM KW6002 (*t* = 2.18, *df* = 22, *P* < 0.05, independent-samples *t*-test).

### Blockade of adenosine A_2A_ receptors was involved in exogenous CGS21680-induced modulation of pallidal firing

**Figure 4:**
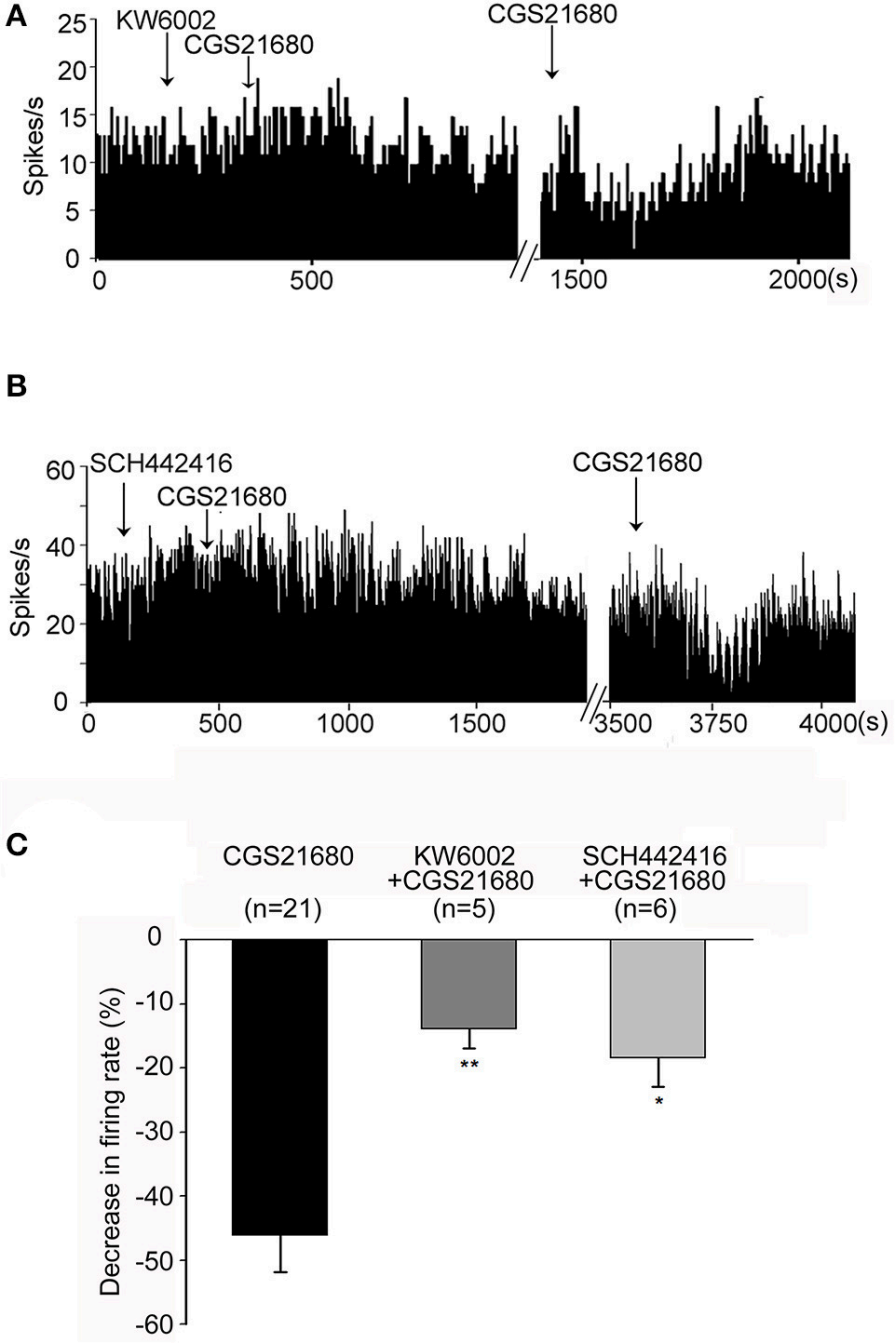
Both KW6002 and SCH442416 blocked CGS21680-induced decrease in firing rate. **(A)** Typical frequency histograms showing that application of 1 μM CGS21680 in the presence of 1 μM KW6002 did not change the firing rate. However, the second time application of CGS21680 alone significantly decreased the firing rate in this neuron. **(B)** Typical frequency histograms showing that 1 μM SCH442416 blocked 1 μM CGS21680-induced decrease in firing rate. **(C)** Pooled data summarizing the effects of both KW6002 and SCH442416 on CGS21680-induced decrease of firing rate. The black bars represent the effects of CGS21680 alone, while the dark gray bars and light gray bars represent effects of CGS21680 together with KW6002 or SCH442416, respectively. ^*^P < 0.05, ^**^P < 0.01, compared to CGS21680 alone group, Mann-Whitney test.

Next two adenosine A_2A_ receptor antagonists KW6002 and SCH442416 were used to further study whether adenosine A_2A_ receptors are involved in CGS21680-induced modulation of pallidal firing rate. As shown in Figure 4A, 1 μM CGS21680 did not change the firing rate significantly in the presence of 1 μM KW6002. The second time application of 1 μM CGS21680 alone decreased the firing rate which indicated that CGS21680-induced inhibitory effect of firing rate was prevented by KW6002. In total 17 pallidal neurons, CGS21680 only decreased the spontaneous firing rate in 5 out of the 17 pallidal neurons in the presence of 1 μM KW6002. The average decrease (13.93 ± 2.96%) of the firing rate was significantly weaker than that in the absence of 1 μM KW6002 (46.08 ± 5.71%, *Z* = 2.83, *P* < 0.01, Mann-Whitney test, Figure 4C). Moreover, SCH442416 also blocked CGS21680-induced decrease of pallidal firing rate (Figures 4B,C). Similar to that of CGS21680-induced inhibitory effects, KW6002 and SCH442416 also blocked the increasing effects of CGS21680 on pallidal firing rate (data not shown).

### Effects of adenosine A_2A_ receptor activation on the spontaneous firing of globus pallidus neurons in 6-OHDA hemi-parkinsonian rats

**Figure 5:**
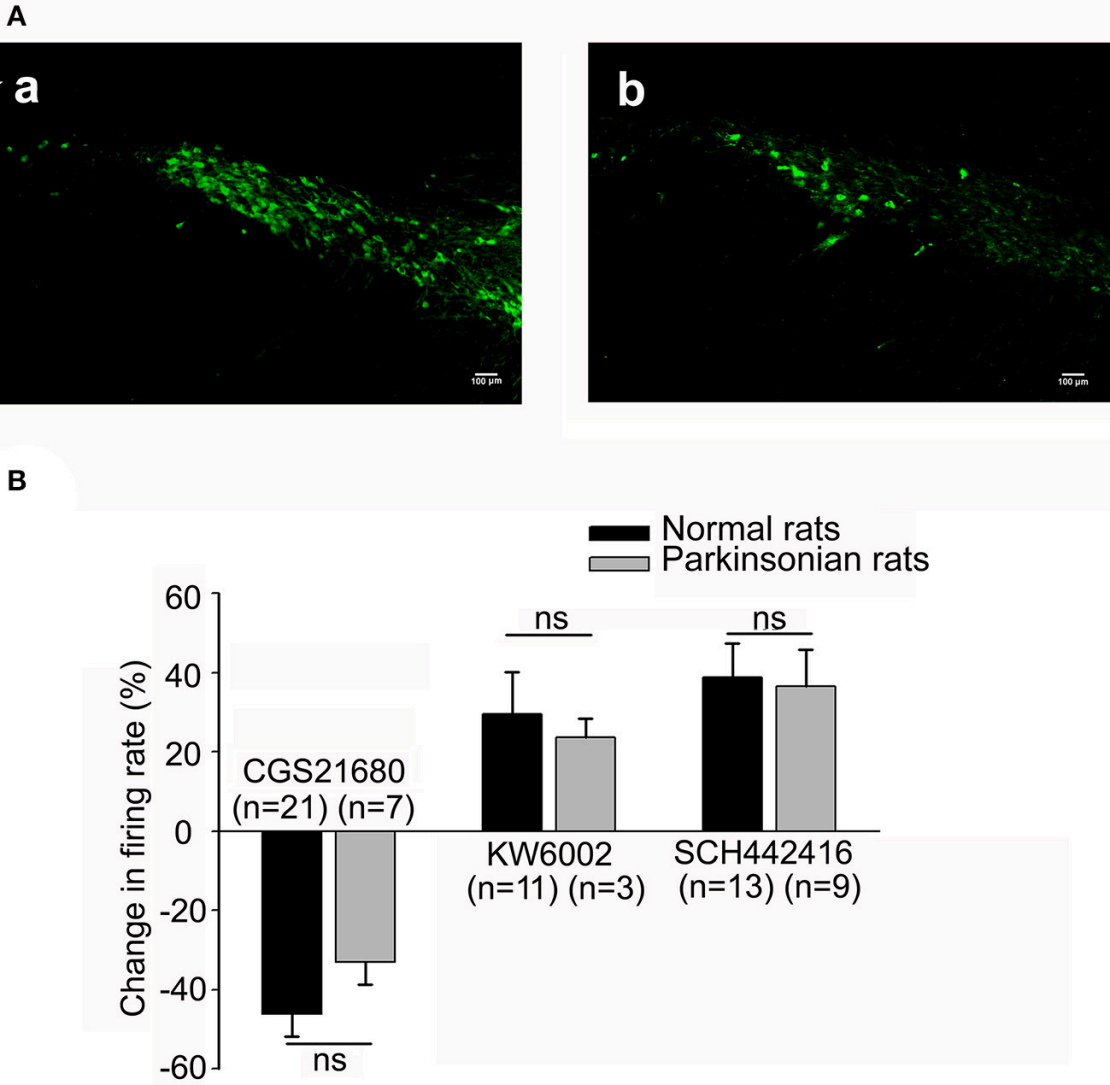
Comparison of the electrophysiological effects of adenosine A_2A_ receptor agonist and antagonist in both normal and parkinsonian rats. **(A)** Confirmation of 6-OHDA hemi-parkinsonian rat model. Fluorescent images revealing tyrosine hydroxylase (TH) immunostaining in the substantia nigra pars compacta (SNc) of both normal (a) and 6-OHDA hemi-parkinsonian (b) rats. Scale bars = 100 μm. **(B)** Comparison of the effects of 1 μM CGS21680, 1 μM KW6002, and 1 μM SCH442416 on the spontaneous firing rate of pallidal neurons between normal (black bars) and 6-OHDA parkinsonian rats (gray bars). *ns*, not significant.

In this study, we further explored the direct modulation of adenosine A_2A_ receptors on the spontaneous pallidal firing in 6-OHDA hemi-parkinsonian rats (Figure 5A). The average basal firing rate of pallidal neurons on lesioned sides of 6-OHDA parkinsonian rats was significantly different from that of normal rats or unlesioned sides of parkinsonian rats (*F* = 8.16, *df* = 2, *P* < 0.01, one-way ANOVA). The average basal firing rate of pallidal neurons on the lesioned side of parkinsonian rats (7.94 ± 0.98 Hz, *n* = 44) was significantly lower than that of normal rats (14.02 ± 1.46 Hz, *n* = 47, *t* = 3.48, *P* < 0.01), as well as that on the unlesioned side of parkinsonian rats (14.33 ± 1.26 Hz, *n* = 40, *t* = 3.51, *P* < 0.01). Concerning the firing patterns, 34.09% (15/44) of the pallidal neurons recorded exhibited low frequency with bursts on the lesioned side of parkinsonian rats, which was more than that of normal rats (7/47, 14.89%, *x*^2^ = 4.57*, df* = 1, *P* < 0.05, Chi-square test).

Furthermore, we observed the effects of micro-pressure ejection of CGS21680 (1 μM) on the spontaneous firing activity of pallidal neurons in 6-OHDA hemi-parkinsonian rats. On the lesioned side, 1 μM CGS21680 significantly decreased the firing rate from 8.14 ± 2.04 to 6.20 ± 1.81 Hz in 7 out of the 19 neurons (*n* = 7, *t* = 6.92, *df* = 6, *P* < 0.001, paired-samples *t*-test). The average decrease was 33.04 ± 5.63% (*t* = 5.99, *df* = 13, *P* < 0.01 compared with that of vehicle ejection, independent-samples *t*-test). In 2 neurons the frequency increased by 40.18 ± 18.74% and in the left 10 neurons the firing rate was not affected (*t* = 1.46, *df* = 16, *P* > 0.05, independent-samples *t*-test). On the unlesioned side, 1 μM CGS21680 also exhibited bidirectional effects on spontaneous discharge of pallidal neurons (data not shown). The CGS21680-induced inhibitory effects on both lesioned and unlesioned sides of parkinsonian rats were not different from that in normal rats (*F* = 0.68, *df* = 2, *P* > 0.05, one-way ANOVA, Figure 5B). Moreover, similar to that of normal rats, 1 μM CGS21680 didn't significantly change the pallidal firing pattern on both lesioned and unlesioned sides of parkinsonian rats (data not shown).

### Effects of endogenous adenosine on the spontaneous firing of globus pallidus neurons through adenosine A_2A_ receptors in 6-OHDA hemi-parkinsonian rats

Previous studies have found that blockade of adenosine A_2A_ receptors exerts therapeutic potential in Parkinson's disease. We further observed the direct regulation of the spontaneous pallidal firing by two selective adenosine A_2A_ receptor antagonists, KW6002 and SCH442416, in 6-OHDA hemi-parkinsonian rats.

We firstly observed the effects of 1 μM KW6002 on the pallidal firing rate in 6-OHDA hemi-parkinsonian rats. On the lesioned side, the average basal firing rate of 14 neurons recorded was 10.04 ± 2.29 Hz. KW6002 tended to increase the spontaneous firing rate in 3 out of the 14 pallidal neurons (basal: 15.42 ± 5.51 Hz; KW6002: 18.57 ± 6.34 Hz, *n* = 3, *t* = 3.66, *df* = 2, *P* = 0.07, paired-samples *t*-test), but there was no statistical difference. The average increase was 23.72 ± 4.59% (Figure 5B). In only one neuron, KW6002 decreased the firing rate with average decrease of 49.75% and in the left 10 neurons, KW6002 did not significantly change the firing rate (*t* = 0.71, *df* = 9, *P* > 0.05, paired-samples *t*-test). On the unlesioned side, the average basal firing rate of 16 neurons recorded was 14.98 ± 2.19. 1 μM KW6002 significantly increased the firing rate by 26.08 ± 9.41% in 8 out of the 16 pallidal neurons (*t* = 2.52, *df* = 14, *P* < 0.05 compared with that of vehicle group, independent-samples *t*-test), while did not change the firing rate in the remaining 8 neurons (*t* = 0.34, *df* = 14, *P* > 0.05 compared with that of vehicle group, independent-samples *t*-test). In addition, the percentage of KW6002 responsive neurons on the lesioned side (28.57%, 4 out of 14) tended to be lower than that in normal rats (55.56%, 15 out of 27), and that on the unlesioned side (50.00%, 8 out of 16), although there was no statistic difference. Further studies revealed that SCH442416 increased the firing rate from 11.31 ± 3.10 Hz to 14.86 ± 3.92 Hz in 9 out of the 21 neurons on the lesioned side (*n* = 9, *t* = 2.74, *df* = 8, *P* < 0.05, paired-samples *t*-test). The average increase was 36.47 ± 10.30% (*t* = 3.42, *df* = 13, *P* < 0.01 compared with that of vehicle group, independent-samples *t*-test). Furthermore, 1 μM SCH442416-induced excitatory effects of pallidal neurons on the lesioned side of parkinsonian rats was not significantly different from that of normal rats (*t* = 0.39, *df* = 20, *P* > 0.05, independent-samples *t*-test, Figure 5B).

### Effects of application of both adenosine A_2A_ receptor antagonist and dopamine D_2_ receptor agonist on the spontaneous firing of globus pallidus neurons in normal and hemi-parkinsonian rats

**Figure 6:**
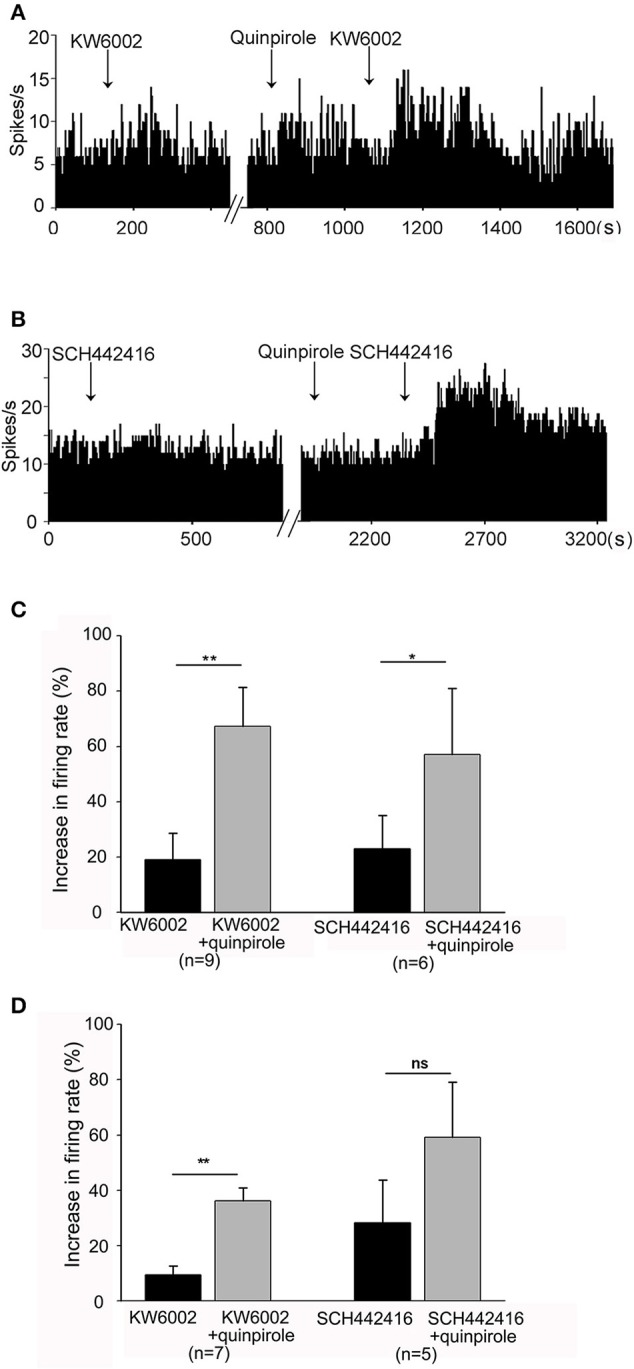
Quinpirole enhanced KW6002 and SCH44216-induced increase in firing rate of pallidal neurons. **(A)** The first time application of 1 μM KW6002 alone increased the pallidal firing rate by 48.86% in this neuron of normal rat. In the presence of 1 μM quinpirole, the second time application of KW6002 significantly increased the firing rate by 78.07% in this neuron. **(B)** Typical frequency histograms showing that quinpirole enhanced 1 μM SCH44216-induced excitation of firing rate in this neuron. **(C)** Pooled data summarizing the effects of quinpirole on KW6002 and SCH44216-induced increase of firing rate in normal rats. **(D)** Pooled data summarizing the effects of quinpirole on KW6002 and SCH44216-induced increase of firing rate in parkinsonian rats. The black bars represent the effects of KW6002 or SCH44216 alone, while the gray bars represent the effects of KW6002 or SCH44216 together with quinpirole. ^*^P < 0.05, ^**^P < 0.01, *ns*, not significant, paired-samples *t*-test.

Next, extracellular recordings were used to further explore the interactions between adenosine A_2A_ receptors and dopamine D_2_ receptors in normal and hemi-parkinsonian rats. Firstly, we observed the relationship between adenosine A_2A_ receptors and dopamine D_2_ receptors in normal rats. Adenosine A_2A_ receptor antagonist was applied first. In the same neuron, this antagonist was applied again in the presence of selective dopamine D_2_ receptor agonist quinpirole. As shown in Figures 6A,**C**, the first-time application of adenosine A_2A_ receptor antagonist KW6002 (1 μM) alone slightly increased the pallidal firing rate by 19.14 ± 9.51% in 9 out of 15 pallidal neurons. However, in the presence of quinpirole (1 mM), the second time application of KW6002 significantly increased the spontaneous firing rate by 67.33 ± 14.09% in the same 9 neurons, which was stronger than that in the absence of quinpirole (*Z* = 2.67, *P* < 0.01, Wilcoxon signed-rank test). In another set of experiment, quinpirole enhanced SCH442416-induced increase of pallidal firing rate from 23.00 ± 12.12% to 57.06 ± 23.90% (*Z* = 2.20, *P* < 0.05, Wilcoxon signed-rank test, Figures 6B,C). Secondly, we explored the relationship of adenosine A_2A_ receptors and dopamine D_2_ receptors in hemi-parkinsonian rats. Similar to that of normal rats, the excitatory effects induced by combination of quinpirole with KW6002 or SCH442416 tended to be stronger than that of adenosine A_2A_ receptor antagonist alone in hemi-parkinsonian rats (Figure 6D).

### Gabaergic neurotransmission was involved in CGS21680-induced modulation of the spontaneous firing of globus pallidus neurons

**Figure 7:**
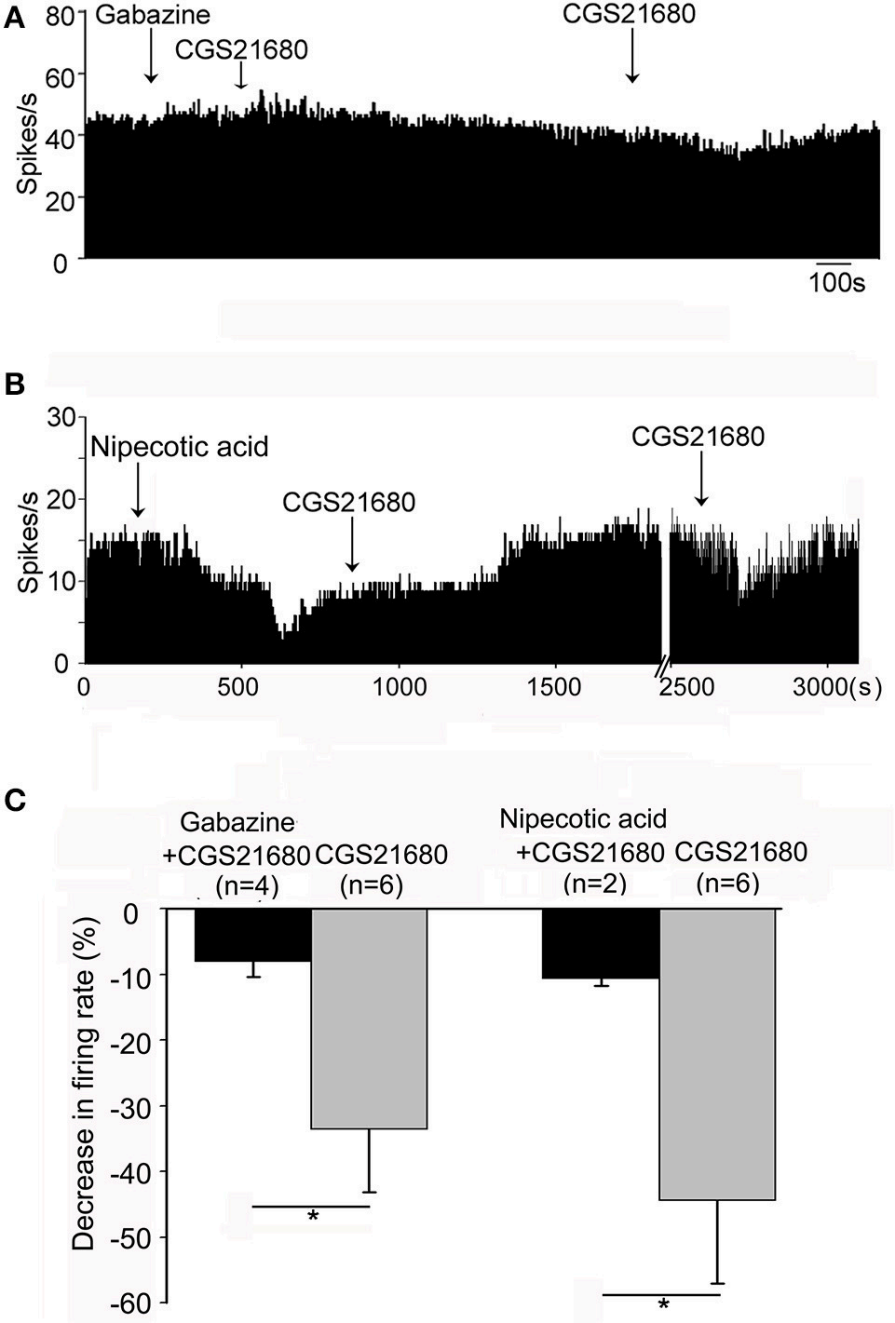
GABAergic transmission was involved in CGS21680-induced inhibition of the firing activity of globus pallidus neurons in normal rats. **(A)** Typical frequency histogram showing that 0.1 mM gabazine blocked CGS21680-induced decrease of firing rate of globus pallidus neuron. **(B)** Typical frequency histogram showing that in the presence of 10 μM nipecotic acid, CGS21680 did not induce clear decrease of firing rate. However, after long time recovery, the second time application of CGS21680 alone decreased the firing rate in the same neuron. **(C)** Pooled data summarizing CGS21680-induced decrease of firing rate in the presence (black bars) and absence (gray bars) of gabazine or nipecotic acid. ^*^P < 0.05, Mann-Whitney test.

The competitive GABA_A_ receptor antagonist (gabazine) and the selective GABA transporter-1 inhibitor (nipecotic acid) were used to further identify the possible involvement of GABAergic neurotransmission in CGS21680-induced decrease of firing rate. As shown in Figures 7A,C, in the presence of gabazine, CGS21680 only decreased the firing rate by 8.04 ± 2.35% in 4 out of the 13 globus pallidus neurons, which was significantly weaker than that of the second time application of CGS21680 in the absence of gabazine (decrease: 33.59 ± 9.59%, *n* = 6, *Z* = 2.35, *P* < 0.05, Mann-Whitney test). In another set of experiment, nipecotic acid (10 μM) significantly decreased the firing rate from 14.73 ± 2.73 Hz to 6.12 ± 2.28 Hz in 17 out of 23 pallidal neurons tested (*n* = 17, *t* = 5.45, *df* = 16, *P* < 0.01, paired-samples *t*-test, Figure 7B). In the presence of nipecotic acid, CGS21680 decreased the firing rate by 10.59 ± 1.15% in only 2 out of the 6 neurons in which the second time application of CGS21680 alone inhibited the firing rate by 44.38 ± 12.62% (*Z* = 2.00, *P* < 0.05, Mann-Whitney test, Figure 7C).

### CGS21680 modulated the spontaneous firing activity of globus pallidus neurons through PKA pathway

**Figure 8:**
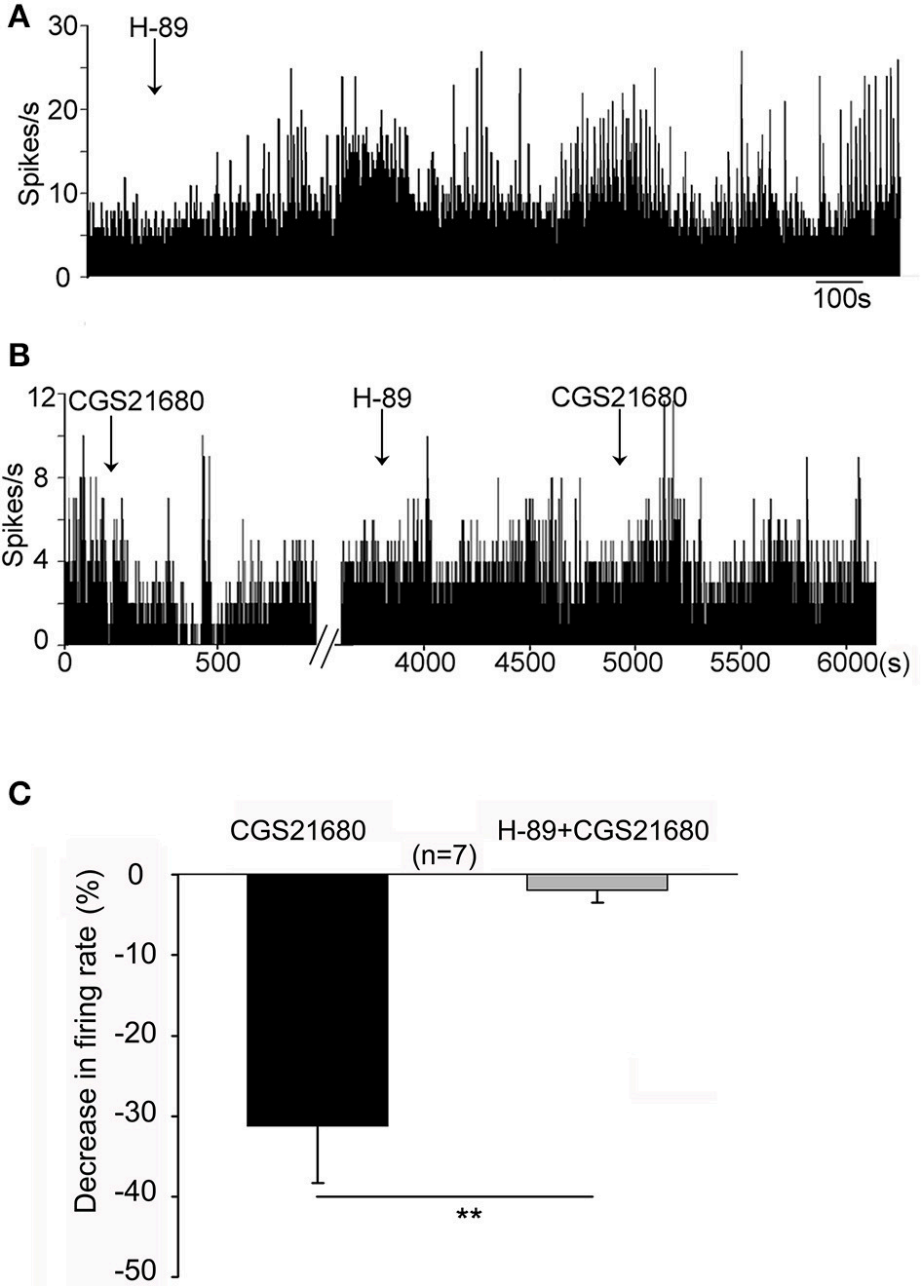
PKA pathway was involved in CGS21680-induced change of pallidal firing activity in normal rats. **(A)** Application of H-89 (10 μM) significantly increased the firing rate. **(B)** The first time application of 1 μM CGS21680 decreased the firing rate. In the presence of H-89, the second time application of CGS21680 did not alter the firing rate in this cell. **(C)** Pooled data showing that H-89 significantly blocked CGS21680-induced decrease of firing rate in pallidal neurons tested. The black bars and gray bars represent the effects of CGS21680 in the absence and presence of H89, respectively. ^**^P < 0.01, Wilcoxon signed-rank test.

It is known that activation of adenosine A_2A_ receptors stimulates G-protein/AC/cAMP/PKA signaling pathway. We determined whether selective PKA inhibitor, H-89, blocked adenosine A_2A_ receptor-induced modulation of firing activity of globus pallidus neurons. In one set of experiment, micro-pressure administration of H-89 (10 μM) significantly increased the spontaneous firing rate from 7.05 ± 1.43 Hz to 13.36 ± 2.65 Hz in 12 out of 15 pallidal neurons tested (*n* = 12, *t* = 3.74, *df* = 11, *P* < 0.01, paired-samples *t*-test, Figure 8A). The average increase was 107.29 ± 26.29%. This effect occurred within 15 min after H-89 injection and lasted over 30 min. In the remaining 3 pallidal neurons, H-89 did not alter the firing rate significantly (*t* = 3.03, *df* = 2, *P* > 0.05, paired-samples *t*-test). In another set of experiments, CGS21680 (1 μM) was applied first to pallidal neurons. In neurons which were inhibited by CGS21680, CGS21680 was applied again in the presence of H-89 (10 μM). As shown in Figures 8B,C, CGS21680 alone decreased the spontaneous firing rate from 11.40 ± 2.15 to 7.78 ± 1.87 Hz in 7 out of 13 pallidal neurons (*n* = 7, *t* = 3.11, *df* = 6, *P* < 0.05, paired-samples *t*-test). The average decrease was 31.22 ± 7.11%. In the presence of H-89, the second time application of CGS21680 did not cause significant change in spontaneous firing rate (1.97 ± 1.53%, *Z* = 3.23, *P* < 0.01 compared with that of CGS21680 alone, Wilcoxon signed-rank test).

### Asymmetrical motor behavior induced by pallidal adenosine A_2A_ receptors in awake normal and 6-OHDA hemi-parkinsonian rats

**Figure 9:**
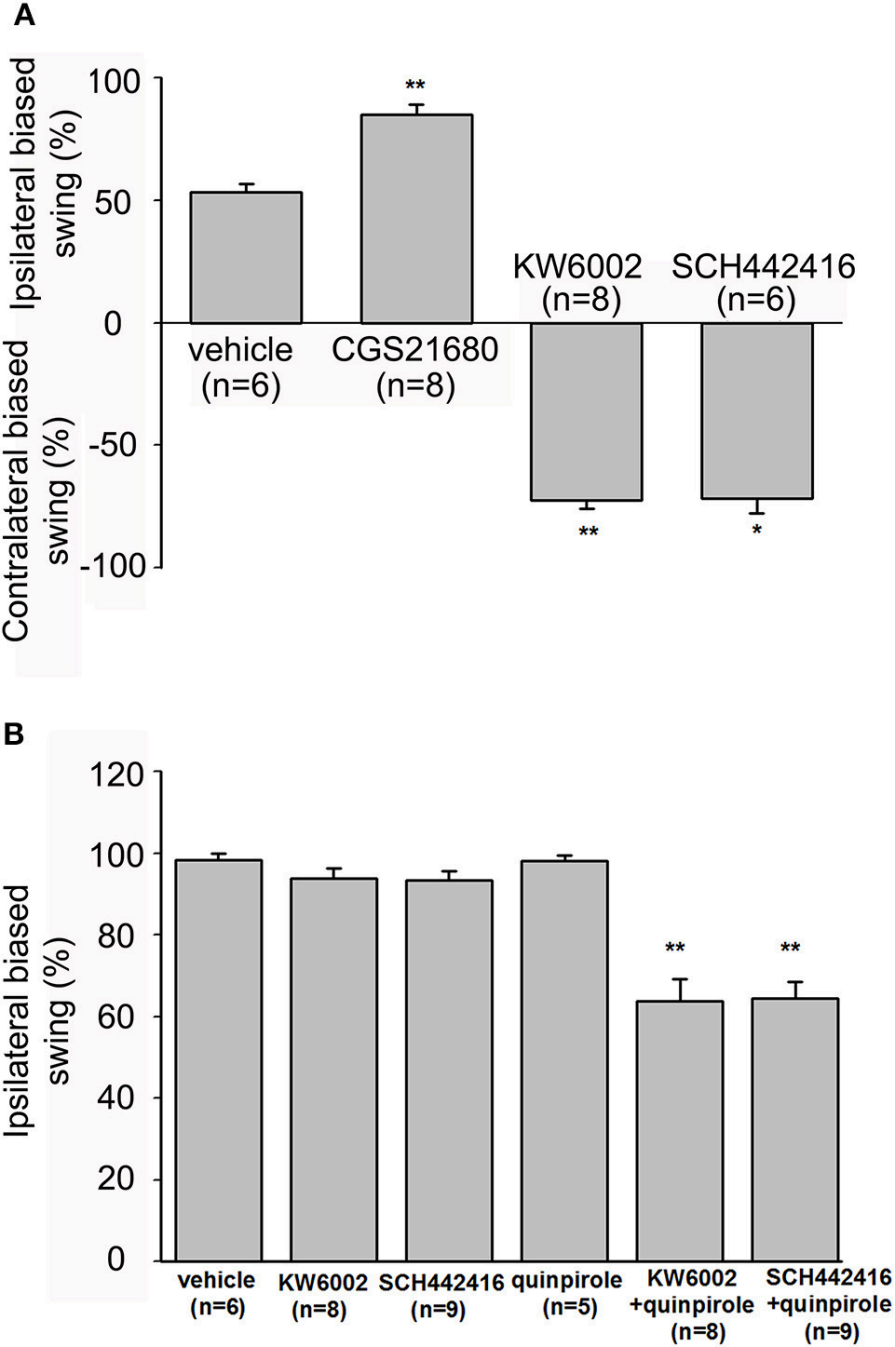
Evaluation of modulatory effects of pallidal adenosine A_2A_ receptors on body asymmetry in awake normal and 6-OHDA hemi-parkinsonian rats using elevated body swing test. **(A)** In normal rats, intrapallidal microinjection of CGS21680 (1 μM) induced ipsilateral-biased swing, while KW6002 (1 μM) and SCH44216 (1 μM) induced contralateral-biased swing. **(B)** In 6-OHDA hemi-parkinsonian rats, unilateral microinjection of KW6002 or SCH44216 into the globus pallidus of lesioned side did not alter 6-OHDA-induced ipsilateral biased swing significantly, while co-application of KW6002 or SCH44216 together with quinpirole (0.05 mg/kg, s.c.) decreased the ipsilateral biased swing. ^*^P < 0.05, ^**^P < 0.01 compared to vehicle (saline) group, Mann-Whitney test.

As adenosine A_2A_ receptors could modulate the firing rate of pallidal neurons at single cellular level, we further explored the behavioral effects of modulating adenosine A_2A_ receptors in the globus pallidus by EBST at the integral level. Firstly, the swing responses induced by adenosine A_2A_ receptor agonist and antagonists in awake normal rats were observed. The rats receiving unilateral vehicle administration displayed unbiased swings (53.33 ± 3.33%, *n* = 6). Unilateral microinjection of CGS21680 (1 μM) into the globus pallidus significantly induced strong ipsilateral bias (85.00 ± 4.23%, *n* = 8, *Z* = 3.13, *P* < 0.01 compared to that of vehicle administration, Mann-Whitney test, Figure 9A). In contrast to that of CGS21680, unilateral microinjection of 1 μM KW6002 and 1 μM SCH442416 exhibited biased swings contralateral to the drug-injection side (72.50 ± 3.13%, *n* = 8, *Z* = 2.81, *P* < 0.01 compared to that of vehicle administration and 71.67 ± 6.00%, *n* = 6, *Z* = 2.06, *P* < 0.05, respectively, Mann-Whitney test, Figure 9A). Secondly, the percentages of swing responses in awake parkinsonian rats were studied. In line with previous finding (Abrous et al., 1998), a strong lesion-induced ipsilateral bias was observed in present unilateral 6-OHDA-lesioned rats (98.95 ± 0.72%, *n* = 19). Unilateral microinjection of vehicle into the globus pallidus of lesioned side did not alter lesion-induced ipsilateral bias (98.33 ± 1.67%, *n* = 6). The percentage of lesion-induced bias was not affected by administration of KW6002 (93.75 ± 2.63%, *n* = 8, *Z* = 1.07, *P* > 0.05, Mann-Whitney test) or SCH442416 (93.33 ± 2.36%, *n* = 9, *Z* = 1.11, *P* > 0.05, Mann-Whitney test). Furthermore, the rats were intrapallidally injected with KW6002 (1 μM) or SCH442416 (1 μM) 30 min after quinpirole administration (0.05 mg/kg, s.c.). Quinpirole alone did not alter lesion-induced ipsilateral bias (98.00 ± 1.49%, *n* = 5). Co-administration of quinpirole with KW6002 or SCH442416 significantly decreased lesion-induced ipsilateral biased swing (63.75 ± 5.32%, *n* = 8, *Z* = 2.99, *P* < 0.01 and 64.44 ± 4.12%, *n* = 9, *Z* = 3.27, *P* < 0.01, respectively, Mann-Whitney test, Figure 9B).

### Expression of adenosine A_2A_ receptors and parvalbumin in the globus pallidus neurons

**Figure 10:**
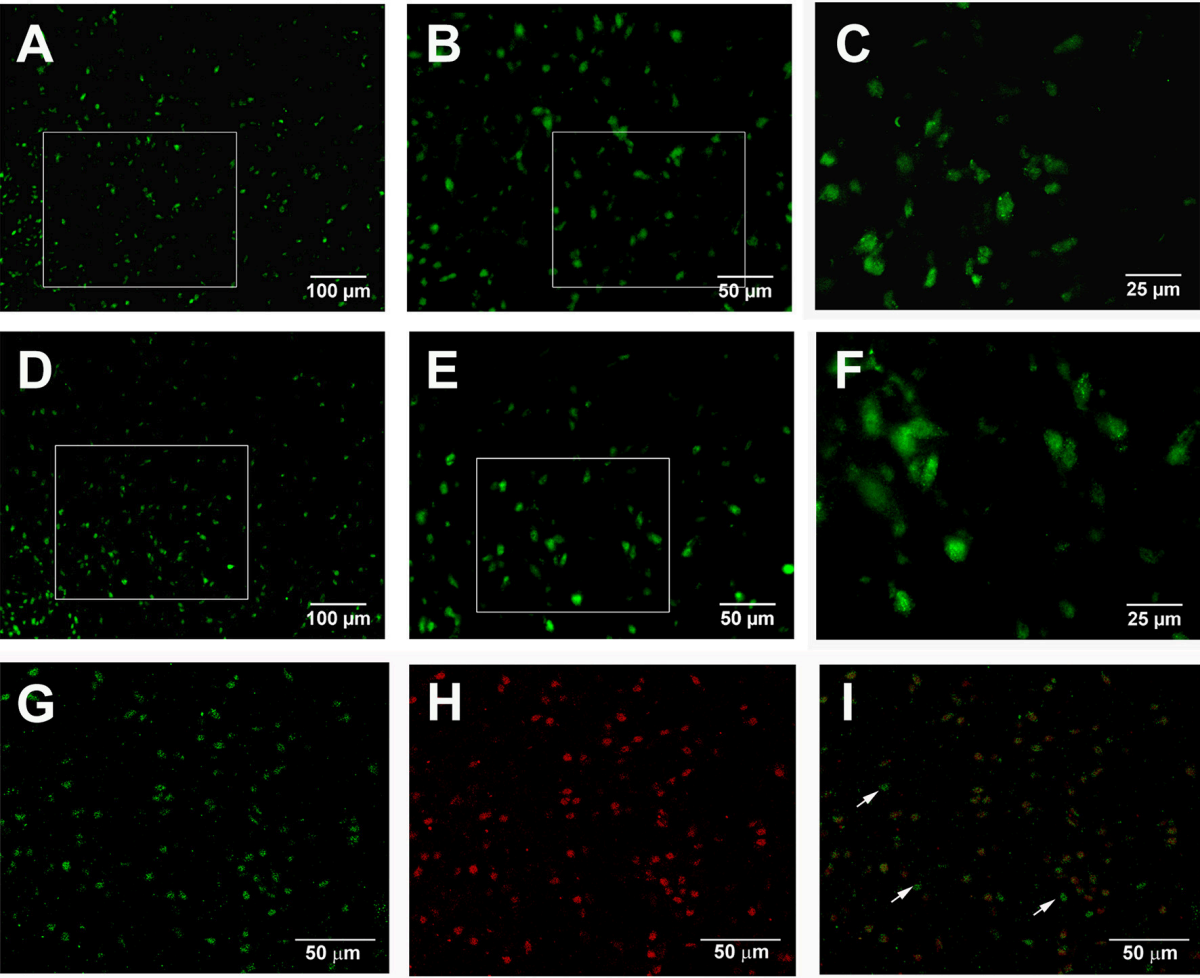
Double immunostaining for adenosine A_2A_ receptors and parvalbumin in the globus pallidus. Fluorescence photomicrographs showing the expression of adenosine A_2A_ receptors in the pallidal neurons in both normal **(A–C)** and 6-OHDA parkinsonian **(D–F)** rats. Confocal laser scanning photomicrographs **(G,H,I)** showing the expression of adenosine A_2A_ receptors (green) and parvalbumin (red), and the overlapping expression (yellow) in normal rats. The arrow indicated non-overlap of parvalbumin and adenosine A_2A_ receptors in this cell. Scale bars = 100 μm in **(A,D)**; 50 μm in **(B–I)**; 25 μm in **(C,F)**.

The present immunostaining showed that adenosine A_2A_ receptors were expressed in the globus pallidus of both normal and 6-OHDA parkinsonian rats. Average number of pallidal adenosine A_2A_ receptor-positive neurons per slice in normal rats was 34.96 ± 1.88, which was not significantly different from that in 6-OHDA parkinsonian rats (36.04 ± 2.39, *Z* = 0.73, *P* > 0.05, Wilcoxon signed-rank test, Figures 10A–F). Moreover, the cellular location of adenosine A_2A_ receptors and parvalbumin in the globus pallidus was studied by using double immunofluorescence labeling. The result showed that adenosine A_2A_ receptors were expressed in both parvabumin-positive and parvabumin-negative neurons (Figures 10G–I).

## Discussion

### Activation of adenosine A_2A_ receptors inhibits the spontaneous firing of pallidal neurons through facilitation of gabaergic neurotransmission

The present electrophysiological results showed that activation of adenosine A_2A_ receptors by CGS21680 mainly exerted inhibitory effects on the spontaneous firing of the globus pallidus neurons which were blocked by adenosine A_2A_ receptor antagonists. It is well-known that the globus pallidus receives dense GABAergic innervation originating from the striatum. Our previous studies have shown that endogenous GABA modulates pallidal firing through GABA_A_ receptors in both rats and mice (Xue et al., 2010; Chen et al., 2013). The bulk of evidence has indicated that presynaptic stimulation of adenosine A_2A_ receptors bidirectionally regulates GABA release from globus pallidus slices in rats. For example, Floran et al. (2005) show that CGS21680 evokes GABA release from rat globus pallidus slices at a wide range of concentration (10 nM to 10 μM). It has been reported that adenosine A_2A_ receptor agonist exerts facilitatory effects on GABA release at lower concentrations, but inhibitory effects at higher concentrations (Mayfield et al., 1993; Dayne Mayfield et al., 1996; Morales-Figueroa et al., 2014). The present *in vivo* extracellular recordings demonstrated that pre-application of GABA_A_ receptor antagonist, gabazine, blocked CGS21680-induced decrease of firing rate suggesting that enhancement of GABAergic neurotransmission may be involved in activation of adenosine A_2A_ receptor-induced inhibition of firing activity in the globus pallidus. GABA transporter-1 (GAT-1) is the prominent transporter in rat globus pallidus. Gonzalez et al. (2006) found that stimulation of adenosine A_2A_ receptors increases GABA level through inhibition of GAT-mediated GABA uptake. Therefore, the high level of extracellular GABA in the globus pallidus may be the major possible mechanism of adenosine A_2A_ receptor-induced inhibition of spontaneous discharge of pallidal neurons. Our *in vivo* electrophysiological studies further revealed that in the presence of the selective GAT-1 inhibitor, nipecotic acid, CGS21680 could not induce inhibitory effect on the spontaneous firing of the globus pallidus neurons. These findings enable us to further identify that blockade of GABA transporter-induced enhancement of GABAergic neurotransmission may be involved in activation of adenosine A_2A_ receptor-mediated inhibition of pallidal neurons. Moreover, the present results suggested that activation of cAMP/PKA pathway was involved in CGS21680-induced modulation of firing activity of pallidal neurons, which was consistent with previous study (Shindou et al., 2002).

### Endogenous adenosine modulates the activity of pallidal neurons through A_2A_ receptors, which may be associated with heteromers with dopamine D_2_ receptors

Present *in vivo* electrophysiological and behavioral studies first demonstrated that application of adenosine A_2A_ receptor antagonists increased the spontaneous firing rate of pallidal neurons and induced contralateral biased swing of rats, suggesting that endogenous adenosinergic system is involved in the regulation of the firing activity of the globus pallidus and motor behavior in normal rats. Adenosine A_2A_ receptors could be divided into two proposed populations based on whether forming heteromers with dopamine D_2_ receptors (Ferre et al., 2008; Orru et al., 2011). The different pharmacological properties of the two antagonists, KW6002 and SCH442416, depend on their affinities to different population of adenosine A_2A_ receptors. KW6002 has high affinity for adenosine A_2A_ receptors forming heteromers with dopamine D_2_ receptors, while SCH442416 shows very low affinity for adenosine A_2A_ receptors co-expressed with dopamine D_2_ receptors. The present electrophysiological study revealed that two populations of adenosine A_2A_ receptors both are involved in endogenous adenosine-induced modulation of pallidal neuronal activity. Moreover, the present electrophysiological results also illustrated that the effects of SCH442416 on pallidal firing (38.78 ± 8.56%) were slightly stronger than that of KW6002 (29.48 ± 10.76%). The percentage of SCH442416-induced excitatory neurons (13 out of 22, 59.09%) was a bit higher than that of KW6002 (11 out of 27, 40.74%). Early studies have demonstrated that anesthesia especially urethane reduces dopamine release (Kelland et al., 1989; Hamilton et al., 1992). Therefore, a possible explanation for the different intensity of the two antagonists may be that anesthetic reduces dopamine release and then inhibits the activity of endogenous adenosine A_2A_ receptor/dopamine D_2_ receptor complex. Moreover, the present study showed that the percentage of KW6002-responsive neurons (4 out of 14, 28.57%) on the lesioned side of hemiparkinsonian rats tended to be lower than that of normal rats (15 out of 27, 55.56%), as well as that on the unlesioned side (8 out of 16, 50.00%). The weaker effect of KW6002 in dopamine-denervated side may be related to the lower extent of dopamine binding to D_2_ receptors. Additionally, Suarez et al. (2016) demonstrates that dopamine depletion reduces dendritic spines of striatal medium spiny neurons expressing dopamine D_2_ receptors and the loss is accompanied with a decrease in synaptic strength. Adenosine A_2A_ receptor-dopamine D_2_ receptor heteromers are selectively localized on the terminals of striatal medium spiny neurons in the globus pallidus (Floran et al., 2005). Whether the weaker effect of KW6002 is related to the morphology changes of synaptic spine remains to be explored.

### Activation of adenosine A_2A_ receptors induces a weak excitation in partial pallidal neurons

The present study also showed that activation of adenosine A_2A_ receptors produced a weak excitation in a few parts of pallidal neurons. In addition to GABAergic innervation, the globus pallidus receives cholinergic innervation from the brainstem pedunculopontine tegmental nucleus (Eid et al., 2016). Nicotine acetylcholine increases spiking rate of pallidal neurons (Rios et al., 2016). It has been reported that activation of adenosine A_2A_ receptors by CGS 21680 facilitates acetylcholine release in some brain regions including hippocampus and striatum (Kurokawa et al., 1994; Ribeiro et al., 1996; Rebola et al., 2002). Although, no morphological study shows the expression of adenosine A_2A_ receptors on cholinergic nerve terminals in the globus pallidus, one may hypothesize that adenosine A_2A_ receptor-mediated possible modulation of acetylcholine release may be responsible for CGS21680-induced weak excitatory effects.

It is well-known that the globus pallidus neurons are diverse in electrophysiology, axonal projections, dendritic morphology, and the expression of molecular markers (Cooper and Stanford, 2000; Benhamou et al., 2012; Mallet et al., 2012; Hernandez et al., 2015; Karain et al., 2015). The CGS21680-induced bidirectional effects (inhibition or excitation) may be associated with the diversity of pallidal neurons. The GABAergic globus pallidus neurons are classificated into parvalbumin-positive and parvalbumin -negative neurons based on the expression of the calcium-binding protein parvalbumin (Saunders et al., 2016). Parvalbumin-positive neurons represent the majority of “prototypic” pallidal neurons which exhibit fast and regular firing spontaneous activity and innervate primarily the subthalamic nucleus. Most parvalbumin-negative neurons are “arkypallidal” pallidal neurons which exhibit slower and more irregular spontaneous activity and project strongly back to dorsal striatum. Based on the classification of firing patterns, the present electrophysiological study showed that CGS21680-induced decrease or increase of firing activity was observed in all the three types of pallidal neurons. Additionally, the present double immunostaining showed that adenosine A_2A_ receptors are located on both parvalbumin-positive and parvalbumin-negative pallidal neurons. Unfortunately, for the technical limitation, we could not label the recorded neurons and further identify the types of pallidal neurons precisely.

### Adenosine A_2A_ receptors are functional in 6-OHDA parkinsonian rats

The present extracellular recordings displayed lower basal firing rate of pallidal neurons after 6-OHDA lesions. However, some studies show inconsistent changes of firing rates in parkinsonian models (Zold et al., 2007; Ellens and Leventhal, 2013). According to Leblois et al. (2006), basal ganglia neurons would not exhibit abnormal firing activity unless extensive dopamine depletion is produced. The present immunostaining indicated that the number of hydroxylase-immunoreactive cells in lesioned substantia nigra pars compacta of hemiparkinsonian rats decreased to about 17%. Therefore, the decrease of pallidal firing rates may be associated with severe dopamine depletion in substantia nigra of hemiparkinsonian rats. Inhibition of the globus pallidus may contribute to impeding voluntary movement in Parkinson's disease through indirect pathway of the basal ganglia circuits, and ultimately inhibiting the activities of thalamic motor nuclei and motor cortex. The present adenosine A_2A_ receptor antagonists-induced increase of discharge frequency indicated that adenosine A_2A_ receptor antagonists may contribute to alleviating motor symptoms in Parkinson's disease by normalizing the firing rate of pallidal neurons. Furthermore, the CGS21680-induced inhibition of pallidal firing rate in present parkinsonian rats is not significantly different from that in normal rats. Previous studies have revealed changes of adenosine A_2A_ receptor expression in discrete brain regions of patients dying with Parkinson's disease as well as Parkinson's animal models (Hurley et al., 2000; Villar-Menendez et al., 2014). For example, Hurley et al. (2000) reported the decreasing level of adenosine A_2A_ receptor mRNA in some striatal regions, increasing in the substantia nigra and no change in any other brain regions examined. Similarly, no clear change of the expression of pallidal adenosine A_2A_ receptors was observed in present morphological study, which may be one of the possible reasons for the present similar electrophysiological results under both normal and parkinsonian states.

### Pallidal adenosine A_2A_ receptors modulate motor behavior in awake rats

As adenosine A_2A_ receptors modulated pallidal firing, we hypothesized that pharmacological manipulation of adenosine A_2A_ receptors in the globus pallidus maybe participate in motor modulation in awake rats. EBST is a simple, sensitive and accurate behavioral test used to evaluate asymmetrical behavior in animals with a unilateral cerebral lesion such as Parkinson's disease, Huntington's disease and ischemic stroke (Borlongan et al., 1995; Baluchnejadmojarad and Roghani, 2004; Tabuse et al., 2010; Ingberg et al., 2015). The asymmetric swing behavior has been attributed to the imbalance of motor control in the basal ganglia circuit. In present study, we observed that unilateral microinjection of adenosine A_2A_ receptor agonist or antagonist into the globus pallidus displayed significant biased swing behavior ipsilaterally or contralaterally, respectively. Unilateral microinjection of adenosine A_2A_ receptor agonist produces hypoactivity of the globus pallidus and then disinhibits the subthalamic nucleus. The thalamocortical activity is suppressed by enhanced GABAergic inhibition from the output nucleus of the basal ganglia. Subsequently, the hypoactivity of ipsilateral motor cortex leads to the imbalance of the activity of bilateral limb muscles. The mechanism of adenosine A_2A_ receptor antagonist-induced contralateral swing is just contrary to that of adenosine A_2A_ receptor agonist. Thus, the present behavioral test suggested that pallidal adenosine A_2A_ receptors are involved in motor regulation. Moreover, the strong ipsilateral bias was observed in present unilateral 6-OHDA-lesioned rats. The possible mechanism may be that dopamine degeneration decreases the activity of motor cortex on the lesioned side of parkinsonian rats which therefore induces imbalance of bilateral movement output. It was reported that tail pinch may further cause dopamine release in the unlesioned striatum (Sindhu et al., 2005). Similar to the above supposition, application of adenosine A_2A_ receptor antagonists in the presence of quinpirole could alleviate 6-OHDA-induced biased swing, which further verified that pallidal adenosine A_2A_ receptors play important roles in the therapy of motor symptoms in Parkinson's disease.

In conclusion, the present study indicated that pallidal adenosine A_2A_ receptors play prominent roles in motor modulation under both healthy and parkinsonian states, which further verified that pallidal adenosine A_2A_ receptor is potentially useful in the treatment of Parkinson's disease. Therefore, more studies will be needed to explore the functions of pallidal adenosine A_2A_ receptors in both heath and disease.

## Ethics statement

This study was carried out in accordance with the recommendations of the University guidelines on animal ethics. The protocol was approved by an Animal Ethics Committee of Qingdao University.

## Author contributions

H-LD performed experiments and wrote the draft. H-LD, YX, XHH, S-YW, CL, and W-FC analyzed the data. H-LD wrote the manuscript. LC designed and supervised the project.

### Conflict of interest statement

The authors declare that the research was conducted in the absence of any commercial or financial relationships that could be construed as a potential conflict of interest.

## Abbreviations

6-OHDA: 6-hydroxydopamine
GABA: γ-aminobutyric acid
PKA: protein kinase A
EBST: elevated body swing test.
